# The role of plant processing for the cancer preventive potential of Ethiopian kale (*Brassica carinata*)

**DOI:** 10.1080/16546628.2017.1271527

**Published:** 2017-01-31

**Authors:** Grace Akinyi Odongo, Nina Schlotz, Corinna Herz, Franziska S. Hanschen, Susanne Baldermann, Susanne Neugart, Bernhard Trierweiler, Lara Frommherz, Charles M. A. P. Franz, Benard Ngwene, Abraham Wahid Luvonga, Monika Schreiner, Sascha Rohn, Evelyn Lamy

**Affiliations:** ^a^ Molecular Preventive Medicine, Institute for Prevention and Cancer Epidemiology, University of Freiburg – Medical Center, Freiburg im Breisgau, Germany; ^b^ Department of Plant Quality, Leibniz Institute of Vegetable and Ornamental Crops Grossbeeren/Erfurt e. V., Großbeeren, Germany; ^c^ Department of Safety and Quality of Fruits and Vegetables, Max Rubner-Institut, Karlsruhe, Germany; ^d^ Department of Microbiology and Biotechnology, Max Rubner-Institut, Karlsruhe, Germany; ^e^ Hamburg School of Food Science, Institute of Food Chemistry, University of Hamburg, Hamburg, Germany

**Keywords:** Aflatoxin B1, African leafy vegetables, anti-genotoxicity, *Brassicaceae*, cancer chemoprevention, glucosinolates, polyphenols, secondary plant metabolites

## Abstract

**Background**: Ethiopian kale (*Brassica carinata*) is a horticulturally important crop used as leafy vegetable in large parts of East and Southern Africa. The leaves are reported to contain high concentrations of health-promoting secondary plant metabolites. However, scientific knowledge on their health benefits is scarce.

**Objective**: This study aimed to determine the cancer preventive potential of *B. carinata* using a human liver *in vitro* model focusing on processing effects on the pattern of secondary plant metabolites and bioactivity.

**Design**: *B. carinata* was cultivated under controlled conditions and differentially processed (raw, fermented, or cooked) after harvesting. Human liver cancer cells (HepG2) were treated with ethanolic extracts of raw or processed *B. carinata* leaves and analyzed for their anti-genotoxic, anti-oxidant, and cytostatic potential. Chemical analyses were carried out on glucosinolates including breakdown products, phenolic compounds, carotenoids, and chlorophyll content.

**Results**: Pre-treatment with *B. carinata* extracts concentration dependently reduced aflatoxin-induced DNA damage in the Comet assay, reduced the production of reactive oxygen species as determined by electron paramagnetic resonance spectroscopy, and induced Nrf2-mediated gene expression. Increasing extract concentrations also promoted cytostasis. Processing had a significant effect on the content of secondary plant metabolites. However, different processing methodologies did not dramatically decrease bioactivity, but enhanced the protective effect in some of the endpoints studied.

**Conclusion**: Our findings highlight the cancer preventive potential of *B. carinata* as indicated by the protection of human liver cells against aflatoxin *in vitro*. In general, consumption of *B. carinata* should be encouraged as part of chemopreventive measures to combat prevalence of aflatoxin-induced diseases.

## Introduction

Sub-Saharan Africa is the region of the world with the highest percentage of chronically malnourished people [1]. The reasons for Africa’s serious problem to feed itself with a sufficient quantity and quality of food are numerous [2], especially with emphasis on agronomic constraints and limitations by locally appropriate processing and cooking techniques [3,4]. Traditional indigenous African leafy vegetables (ALVs) are rich in nutrients and health-promoting secondary plant metabolites with suggested preventive effects against non-communicable diseases. This is one of the main reasons why consumption of traditional ALVs presently gains increasing popularity in Africa. However, scientific knowledge on the efficacy of their potential health benefits is unacceptably low. One of the most promising plants in this context is *Brassica carinata*, commonly known as Ethiopian kale or Ethiopian mustard in English, or by the local name *yabesha gomen* in Amharic language. It is cultivated in the Ethiopian highlands as oil seed and leafy vegetable. It is also consumed in East and Southern Africa and less in Western and Central Africa, often accompanying starchy staples. Leaves and seeds are rich in nutrients and are reported to contain high concentrations of glucosinolates, especially 2-propenyl glucosinolate (sinigrin), as well as phenolic compounds [5–8]. These secondary plant metabolites have considerably contributed to the concept of cancer prevention and control in the Western world [9] and could account for a health-promoting effect also of *B. carinata*. Its leaves and tender stems are eaten raw in salad, but much more commonly they are boiled or pickled. Processing might significantly affect the biochemical properties and thus change the bioavailability of such secondary plant metabolites [10,11]. Their rapid loss due to long cooking procedures has been described before [12].

The present study was conducted to screen and evaluate the cancer preventive potential of extracts from *B. carinata* leaves. In particular, it addressed whether food processing (fermentation or cooking) impacts the plants´ potential to protect against aflatoxin B1 (AFB1), a mycotoxin which threatens the health of an estimated 4.5 billion people worldwide [13] and which is the most potent naturally occurring chemical liver carcinogen [14]. Although the exact mechanism for the process of AFB1-mediated carcinogenesis is not known, its conversion into the active, genotoxic AFB1-8,9-epoxide by phase I enzymes of the liver xenobiotic metabolism (mainly CYP3A4) is of absolute relevance [15]. Further, activation of oxidative stress parameters during hepatocarcinogenesis induced by AFB1 was reported [16]. Consequently, the potential to i) interfere with the genotoxicity of AFB1 in metabolically competent liver cells, ii) act against oxidative stress, and iii) trigger cytostasis to remove malignant transformed cells were addressed in this study. Identification and quantification of the relevant health-related secondary plant metabolites present in the different extracts were also made in this context.

## Materials and methods

### Chemicals

Dulbecco’s Modiﬁed Eagle’s Medium (DMEM), fetal calf serum (FCS), trypsin 10x (25 mg/mL), trypsin-EDTA 10x (5 mg/mL and 2.2 mg/mL) and phosphate buffered saline (PBS, without Ca and Mg), L-glutamine, and penicillin/streptomycin (P/S) solution were purchased from Gibco™, Life Technologies GmbH (Darmstadt, Germany). Krebs HEPES buffer (KHB), deferoxamine methanesulfonate (DFO), diethyldithiocarbamic acid sodium (DETC), and 1-hydroxy-3-methoxycarbonyl-2,2,5,5-tetramethylpyrrolidine (CMH) were purchased from Noxygen Science Tranfer & Diagnostics GmbH (Elzach, Germany). Triton-X 100 was purchased from Carl Roth GmbH & Co. KG (Karlsruhe, Germany), dimethyl sulfoxide (DMSO; purity > 99 %) was purchased from Applichem GmbH (Darmstadt, Germany). Absolute ethanol (EtOH), hydrochloric acid (37 %), trypan blue, aflatoxin B1 (purity ≥ 98 %), menadione and ethidium bromide and DEAE-Sephadex A-25 were purchased from Sigma-Aldrich Chemie GmbH (Taufkirchen, Germany). Low melting point agarose (LMPA) and normal melting point agarose (NMPA) were purchased from Serva GmbH (Heidelberg, Germany). Arylsulfatase, isolated from *Helix pomatia*, was purchased from Roche Diagnostics GmbH (Mannheim, Germany).

The following chemicals and reagents were used for chemical analyses: methanol (99.95%), ammonium acetate was purchased from Carl Roth GmbH & Co. KG(Karlsruhe, Germany); tetrahydrofuran (99.7%) was purchsed from VWR International GmbH (Darmstadt, Germany); methyl *tert*-butyl ether (99.8%) was purchased from Chemsolute Th. Geyer GmbH & Co. KG (Renningen, Germany); dichloromethane (99.9%), isopropanol (99.95%), and zeaxanthin were purchased from CaroteNature GmbH (Ostermundigen, Switzerland) and β-carotene, lutein, and chlorophyll *a* and *b* from Sigma-Aldrich Chemie GmbH (Taufkirchen, Germany). Chlorogenic acid, quercetin 3-*O*-glucoside, kaempferol 3-*O*-glucoside, and isorhamnetin-3-*O*-glucoside were purchased from Carl Roth GmbH and Co. KG (Karlsruhe, Germany).

### Plant material

**Figure 1.**
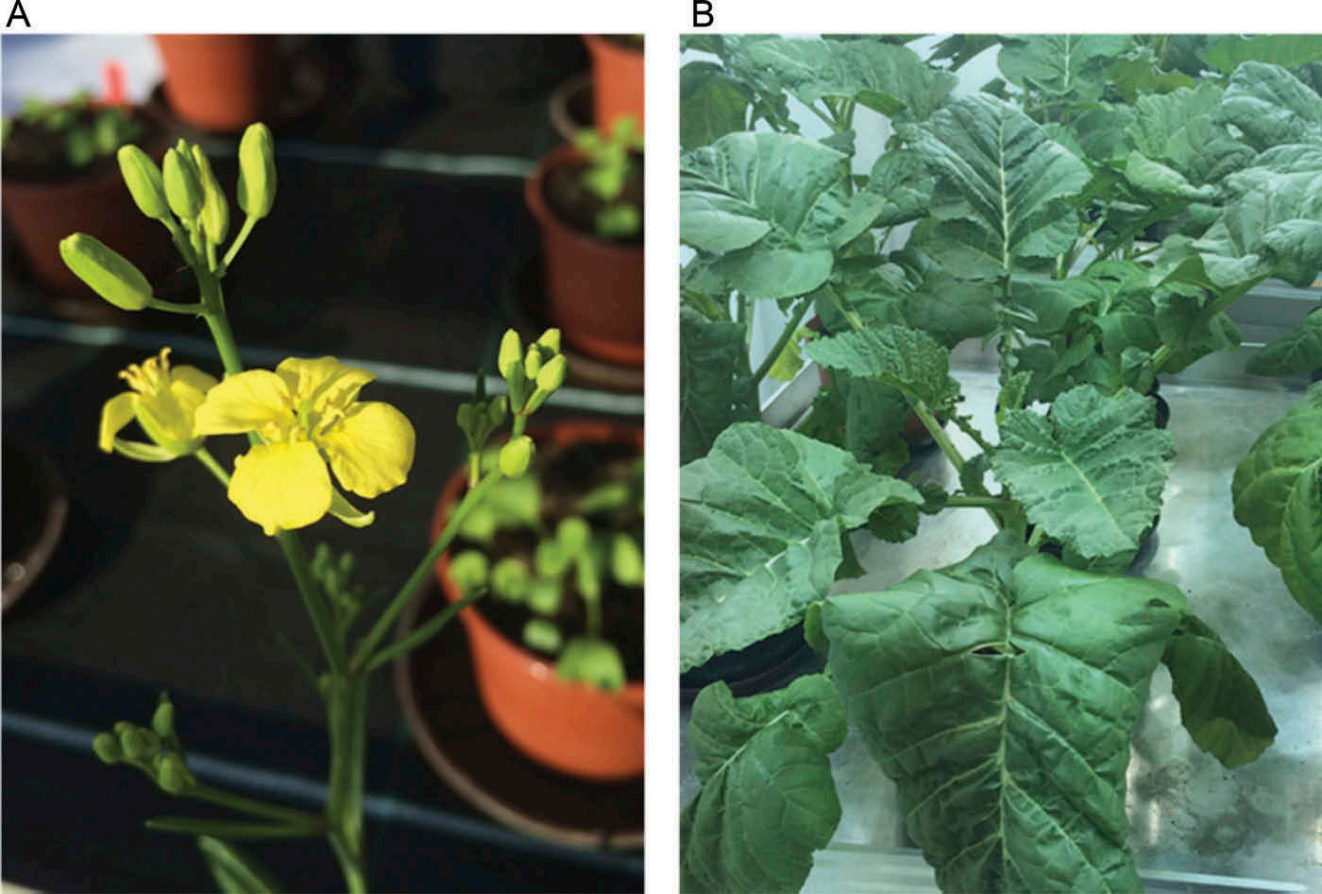
Inflorescences (A) and leaves (B) of *B. carinata.*

Fully developed leaves of *Brassica carinata* A. Braun (Figure 1) were investigated. Seeds of *B. carinata* were provided by the World Vegetable Center (AVRDC). The plants were cultivated at the Max Rubner Institute, Federal Research Institute of Nutrition and Food (location A; Karlsruhe, Germany; at 49° latitude South, 8° longitude East and altitude 116 m above sea level), and at the Leibniz Institute of Vegetable and Ornamental Crops Großbeeren/Erfurt e. V. (location B; Großbeeren, Germany; at 52° latitude North, 13° longitude East and altitude 43 m above sea level).

At location A, *B. carinata* was cultivated using standard potting substrate (Gramoflor) with the following specifications: pH (CaCl_2_) 5.8; N (CaCl_2_ – mg/L) 140; P_2_O_5_ (Cal – mg/L) 160; K_2_O (Cal – mg/L) 180. The plants were grown in a climatic chamber for four weeks at day/night temperatures of 25/20°C, relative humidity of 40/70% and light for 12.5 h. After four weeks, plants were transferred to the greenhouse and kept there under ambient temperature (according to seasonal influences up to 40°C in the summer) and humidity for three weeks.

At location B, seeds of *B. carinata* were sown in 5 L plastic pots containing standard potting substrate (Einheitserdewerke Werkverband e.V., Sinntal-Altengronau, Germany) with the following specifications: pH (CaCl_2_) 5.8; KCl (g/L) 2; N (CaCl_2_ – mg/L) 340; P_2_O_5_ (Cal – mg/L) 380; K_2_O (Cal – mg/L) 420. The plants were grown in a greenhouse for seven weeks after germination with sufficient irrigation. During this period, average day/night temperature was 21/18.6°C respectively with an average air humidity of 54%.

At harvest, fully developed leaves were collected in triplicate, each replicate consisting of 200 g of fresh leave material pooled from about 10 plants. The leaves were then either immediately freeze-dried or further processed.

### Processing of plant material

For fermentation, the freshly harvested leaves (location A) were used for submerged fermentation in 10 L crock pots typically used in Germany for Sauerkraut fermentation as previously described [17]. The leaves were washed with tap water and 700 g of leaves were fermented in 2.1 L of a 2.5% brine solution. The fermentation was inoculated with 1 x 10^7^ CFU/mL of each of the starter bacteria *Lactobacillus plantarum* BFE 5092 and *Lactobacillus fermentum* BFE 6620, and left to ferment at 25°C for 6 days. This fermentation was followed by removal of the brine, weighing of the plant material, freeze drying, and grinding to a fine powder.

For thermal treatments (cooking), the freshly harvested leaves (location B) were sliced to 1 cm pieces with a kitchen knife, and immersed in 100 mL of boiling water. They were cooked (simmered) for 20 min and then drained using a sieve. The drained samples were immediately cooled on ice and then frozen. They were freeze dried, and ground to a fine powder.

### Preparation of ethanolic plant extracts

The freeze dried plant material was extracted with 70% EtOH at a 1:10 ratio, vortexed for 2 min and then placed in a sonicator water bath at 50°C for 30 min. The extract was strained through gauze and filter sterilized using a 0.22 µm MillexR syringe-driven filter unit (fast flow and low binding Millipore). The resulting stock solution had a concentration of 100 mg/mL. For exposure, serial dilutions were prepared and finally diluted at 1:100 in culture medium (max. concentration of solvent: 0.7%). For the Nrf2 reporter gene assay, stock extracts were evaporated under a nitrogen stream, and resuspended in 7% EtOH. Serial dilutions were prepared and then finally diluted at 1:10 in culture medium (max. concentration of solvent: 0.7%). For each independent cell culture experiment extracts were freshly prepared from the respective plant powders and used within two hours. For chemical analyses one extract of each plant powder was freshly prepared.

### Cell lines and culture conditions

The HepG2 cell line (ACC-180) was obtained from the *German Collection of Microorganisms and Cell Cultures* (DSMZ; Braunschweig, Germany). The cells were cultured in DMEM supplemented with 15% FCS and 1% penicillin/streptomycin solution, and incubated in a 95% humidified incubator at 37°C and 5% CO_2_. The recombinant ARE reporter–HepG2 cell line designed to monitor the Nrf2 antioxidant response pathway was purchased from BPS Bioscience, Inc. (San Diego, USA) (60513-GVO-BPS) and cultured as stated for HepG2 cells in the presence of 600 µg/mL geneticin as selection antibiotic.

### Comet assay (single cell gel electrophoresis)

The alkaline Comet assay to detect anti-genotoxic activity of *B. carinata* was performed according to Lamy et al. [18], with few modifications. HepG2 cells pretreated with the ethanolic plant extracts for 24 h were washed twice with PBS and thereafter exposed to 10 µM AFB1 or 0.1% DMSO for another 24 h. Then, cells were harvested and prepared for Comet assay analysis. Analysis was done with a Leica fluorescence microscope (Leica DMLS; excitation filter; BP 546/10 nm; barrier filter: 590 nm) connected to an image analysis system (Comet 5.5, Optilas GmbH, München, Germany), with 100 cells per slide being systematically screened. The indicator of DNA damage was percent tail DNA.

### Electron paramagnetic resonance (EPR) spectroscopy

EPR spectroscopy equipped with temperature and gas controller Bio III (Noxygen) was used to detect the production of reactive oxygen species (ROS) in the HepG2 cells. The protocol described by Lamy et al. [19] was adopted with few modifications. HepG2 cells were pretreated with serial dilutions of ethanolic extracts of *B. carinata* or 0.7% ethanol (solvent) for 24 h, washed twice with PBS, and thereafter exposed to 200 µM menadione or 0.1% DMSO (solvent) for 30 min. The cells were then washed with pre-warmed Krebs-HEPES buffer (KHB) followed by 30 min incubation at 37°C with the high cell permeable spin probe 100 µM CMH in KHB supplemented with 25 µM DFO and 5 µM DETC. Supernatants were transferred to new reaction tubes and kept on ice. EPR spectra were measured in 50 µL glass capillaries. For each sample 10 scans were done.

### Nrf2 antioxidant pathway reporter gene assay

Reporter gene activity was analyzed according to the manufacturer’s instructions using the ONE-Glo™ Luciferase Assay System (Promega GmbH, Mannheim, Germany). In brief, HepG2-ARE cells were seeded in 96 well plates (4*10^4^ cells/well) and immediately exposed to the *B. carinata* extracts. After incubation for 18 h, cells were lysed and luminescence was measured 15 min after substrate addition using an Infinite M200 microplate reader (Tecan Group Ltd, Männedorf, Switzerland).

### Cytotoxicity and cytostatic activity

HepG2 cells were seeded in 12 well plates (10^5^ cells/well), incubated at 37°C/95% humidity at 5% CO_2_ for 48 h and then exposed to serial dilutions of the ethanolic plant extracts. Cytotoxicity and cytostasis were assessed after 48 h treatment using the trypan blue dye exclusion test. Cytotoxicity was determined using the equation

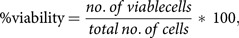

where viable cells are not stained with the dye and the total number of cells is the sum of viable cells plus cells stained with the dye (= non-viable cells). Cytostatic activity was determined by comparing the total numbers of the extract-treated cells with the respective solvent control.

### Chemical analysis

#### Glucosinolates and their breakdown products

Glucosinolates were analyzed after a slightly modified protocol according to Hanschen et al. [20]. Briefly, 0.1 mL of ethanolic plant extracts were loaded onto DEAE-Sephadex A-25 ion-exchanger columns, desulfated using aryl sulfatase and desulfo-GLSs were eluted with deionized water. Desulfo-GLSs were separated using an acetonitrile/water gradient and qualified and quantified by an Agilent 1290 Infinity UHPLC System as reported previously [20]. For analysis of glucosinolate breakdown products, ethanolic fractions were extracted after addition of 2 mL of water according to the protocol of Witzel and co-workers and breakdown products were quantified using gas chromatography-mass spectrometry (GC-MS) as reported previously [21].

#### Phenolic compounds

Phenolic compounds were analyzed according to Schmidt et al. [22] with minor modifications. Compound composition (including hydroxycinnamic acid derivatives and flavonoid glycosides) and concentrations of the ethanolic plant extract were determined using a series 1100 HPLC (Agilent Technologies, Waldbronn, Germany) equipped with a degasser, binary pump, autosampler, column oven, and photodiode array detector. An Ascentis® Express F5 column (150 mm × 4.6 mm, 5 µm, Supelco) was used to separate the compounds at a temperature of 25°C. Eluent A was 0.5% acetic acid, and eluent B was 100% acetonitrile. The gradient used for eluent B was 5–12% (0–3 min), 12–25% (3–46 min), 25–90% (46–49.5 min), 90% isocratic (49.5–52 min), 90–5% (52–52.7 min), and 5% isocratic (52.7–59 min). The detection was conducted at a flow rate of 0.85 mL min^−1^ and wavelengths of 280 nm, 320 nm, 330 nm, 370 nm, and 520 nm. The hydroxycinnamic acid derivatives and glycosides of flavonoids were identified as deprotonated molecular ions and characteristic mass fragment ions according to previously described methods [22,23] by HPLC-DAD-ESI-MS^n^ using a Bruker amazon SL ion trap mass spectrometer in negative ionization mode. Nitrogen was used as the drying gas (10 L min^−1^, 325°C) and the nebulizer gas (40 psi) with a capillary voltage of −3500 V. Helium was used as the collision gas in the ion trap. The mass optimization for the ion optics of the mass spectrometer for quercetin was performed at *m/z* 301 or arbitrarily at *m/z* 1000. The MS^n^ experiments were performed in auto up to MS^3^ in a scan from *m/z* 200–2000. Standards (caffeoylquinic acid [chlorogenic acid], quercetin 3-*O*-glucoside, kaempferol 3-*O*-glucoside, and isorhamnetin-3-*O*-glucoside; Roth, Karlsruhe, Germany) were used for external calibration curves in a semi-quantitative approach.

#### Carotenoids and chlorophylls

Carotenoids and chlorophylls were analyzed on an Agilent Technologies 1290 Infinity UHPLC coupled with an Agilent Technologies 6230 QTOF LC/MS as described by Mageney et al. [24]. Briefly, the separation was performed on a C30-column (YMC Co. Ltd Japan, YMC C30, 100 × 2.1 mm, 3 µm) and mixtures of methanol, methyl *tert*-butyl-ether, and water in different volume ratios (solvent A: 81/15/4 and solvent B: 6/90/4, both 20 mM ammonium acetate) were used as mobile phases at a flow rate of 0.2 mL min^−1^. Identification was achieved by co-chromatography with references substances. External standard calibration curves were used for quantification by dose–response curves.

### Data analysis

All data were analyzed using Graph Pad Prism 6 (GraphPad Software Inc., San Diego, USA). Results of the cell culture experiments are presented as means of at least three independent experiments. Differences were considered significant at p ≤ 0.05 (*), p ≤ 0.01 (**), p ≤ 0.001 (***), p ≤ 0.0001 (****). Statistical significance was assessed using one-way analysis of variance (ANOVA) followed by Dunnett’s multiple comparisons test.

## Results

### *B. carinata* reduces afb1-induced DNA damage

The anti-genotoxic effect of *B. carinata* on human liver cancer (HepG2) cells was determined using the Comet assay. The parameter percent tail DNA (% tail DNA) was used to quantify DNA damage. For better comparisons, all results were calculated in relation to AFB1-treated cells, which were set to 100%. On average, 10 µM AFB1 induced a DNA damage of 9.23% tail DNA which is almost 600% of the solvent control (1.54% tail DNA).

**Figure 2.**
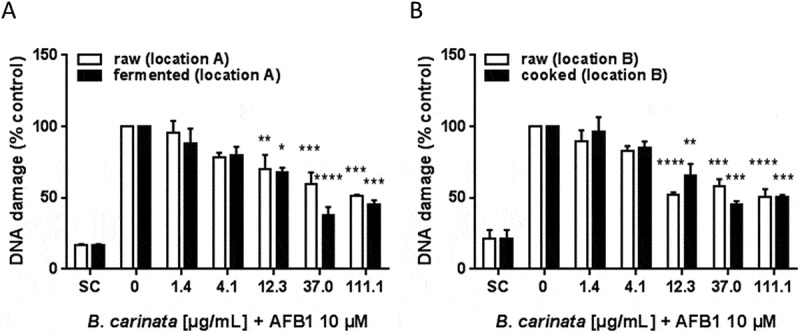
Anti-genotoxic potential of ethanolic extracts of raw and processed B. carinata. DNA damage in AFB1-treated cells is shown as percent of control. Data are means ± SEM of three independent experiments. Asterisks indicate statistically significant differences between the respective treatment and the positive control (= without *B. carinata* extract). SC = solvent control (0.1% DMSO).

Results showed that in general pre-treatment of the cells with the ethanolic plant extracts reduced AFB1-mediated genotoxicity (Figure 2) independent from the processing procedure in a concentration-dependent way. Interestingly, the raw extract from location A (Figure 2(A)) showed a maximum reduction of AFB1-induced genotoxicity of 48.4% at the highest concentration tested (111.1 µg/mL), while the fermented extract showed a maximum reduction of 61.8% already at 37.0 µg/mL. The raw extract from location B (Figure 2(B)) reduced AFB1-induced genotoxicity by 49.1% at the highest concentration tested, which was as high as for the cooked extract. Taken together, fermentation increased and cooking did not impact the anti-genotoxic potency of *B. carinata* leaves.

### Antioxidant activity and induction of ARE/Nrf2-mediated gene expression by ethanolic *B. carinata* plant extracts

In this assay, menadione was used for induction of reactive oxygen species (ROS); a concentration-dependent induction was observed by EPR spectroscopy in the range between 50 to 200 µM menadione, exposed for 30 min (data not shown). At 200 µM, a significant increase of 500% in ROS production as compared to control was detected, and was subsequently used in the experiments at this concentration for challenge studies.

**Figure 3.**
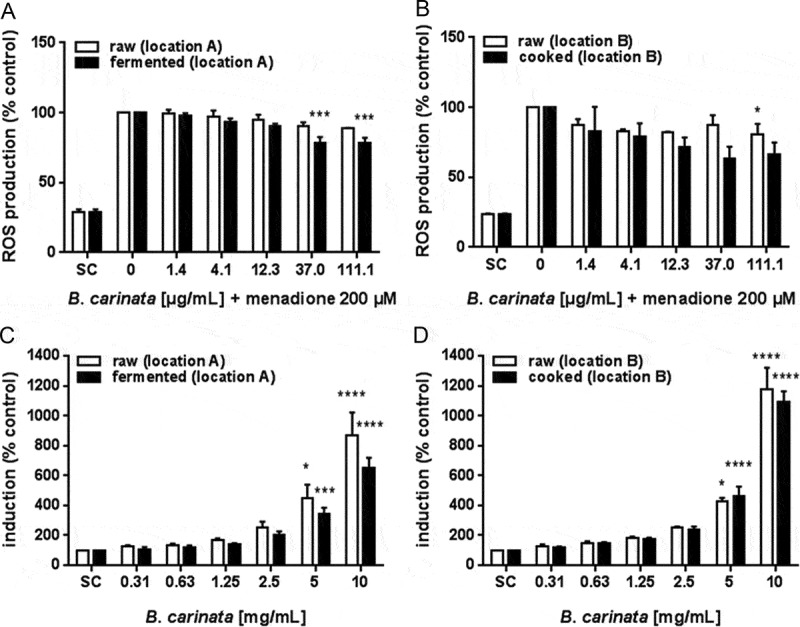
Anti-oxidative potential of ethanolic extracts of raw and processed B. carinata. A and B) ROS production as determined by the EPR method in response to 200 µM menadione in cells pre-treated with *B. carinata* extracts. C) and D) Induction of ARE/Nrf-2-mediated gene expression in cells treated with *B. carinata* extracts. Data are means ± SEM of three independent experiments expressed as percent of control. Asterisks indicate statistically significant differences between the respective treatment and the positive control (= without *B. carinata* extract; A, B) or the SC (C, D). SC = solvent control (0.7% EtOH).

At the highest concentration tested, the raw *B. carinata* extract showed an 11.5% (location A) and 19.7% (location B) reduction of menadione-triggered ROS production in HepG2 cells. At the same concentration, the fermented extract showed a stronger reduction of 21.5%, while the cooked extract blocked menadione-triggered ROS production by 33.3% (Figure 3(A,B)).

Further, all *B. carinata* extracts were able to induce ARE/Nrf2-mediated gene expression in a concentration-dependent manner (Figure 3(C,D)). At the lowest concentration, raw material induced ARE/Nrf2-mediated gene expression by 23% (location A) to 29% (location B). This induction was seen with fermented extracts at 630 µg/mL. At the highest concentration tested in this assay, the raw extracts from location A and B showed an increase of more than 800% and 1180% in ARE/Nrf2-mediated gene expression, respectively. Compared to that, the fermented extract increased gene expression by 650% (Figure 3(C)) whereas the cooked extract showed an induction of 1090 % (Figure 3(D)). Taken together, fermentation as well as cooking increased the capacity of the plant extract to protect against ROS production. Fermentation, but not cooking, showed a tendency to reduce the ability of the plant extract to activate ARE/Nrf2-mediated gene expression.

### Cytotoxicity and induction of cytostasis by ethanolic plant extracts of *B. carinata*

**Figure 4.**
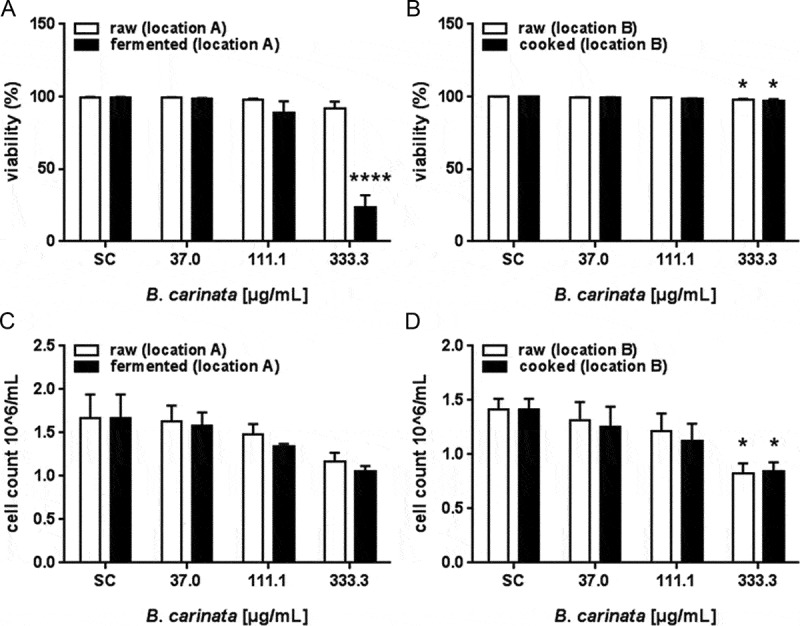
Cytotoxic (A,B) and cytostatic (C, D) potential of ethanolic extracts of raw and processed B. carinata. Data are means ± SEM of three independent experiments. Asterisks indicate statistically significant differences between the respective treatment and SC. SC = solvent control (0.7% EtOH).

Cells treated with fresh and cooked ethanolic extracts of *B. carinata* showed only marginal reduction in viability at the highest concentration tested (Figure 4(A,B)). The fermented extract significantly reduced viability by 76% compared to control cells at the highest concentration (Figure 4(A)). The cytostatic activity of *B. carinata* increased concentration-dependently, but was not influenced by plant processing (Figure 4). At a concentration of 333.3 µg/mL, the extracts reduced the cell number by about 30% (location A; Figure 4(A)) to 40% (location B; Figure 4(B)).

**Figure 5.**
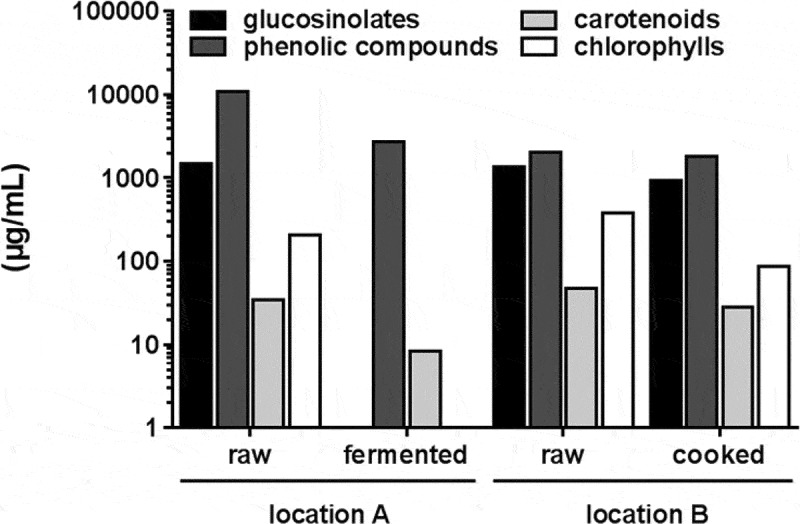
Total content of secondary plant metabolites in ethanolic extracts of raw, fermented, and cooked B. carinata. Results are presented in µg/mL on a logarithmic scale. The first raw and fermented extracts are from location A, while the second raw and cooked extracts are from location B.

### Composition of secondary plant metabolites in raw and processed *B. carinata*

Ethanolic extracts of raw, fermented, and cooked *B. carinata* leaves were analyzed for their glucosinolate, phenolic compound (especially flavonoids), carotenoid, and chlorophyll content (Figure 5 and supplementary Tables S1–S3). The different contents of the secondary plant metabolites in the raw plant material between both locations are probably due to the location-specific environmental conditions during plant cultivation, as was also documented in other studies, e.g. [25].

Sinigrin was the dominant glucosinolate detected in ethanolic extracts of raw *B. carinata*. Upon processing, the concentration declined by 37% in cooked extract and no glucosinolates were present in the extract of the fermented samples (Table S1). With regard to glucosinolate breakdown products, only very low amounts of allyl isothiocyanate were present in the ethanolic *B. carinata* extracts; no allyl isothiocyanate was detected in extracts of cooked material, while 1.0 (location A) to 0.22 µg/mL (location B) was present in the raw material, and 3.3 µg/mL was found in the extract of the fermented material.

Phenolic compounds were reduced during fermentation by 75%. However, there was a structure-dependent degradation. While small hydroxycinnamic acid derivatives (e.g. caffeoylquinic acid) were more stable under fermentation conditions, the more complex ones were degraded more easily. Most flavonol glycosides were decreased by more than 50%. There were some stable compounds such as quercetin-3-*O*-sophoroside-7-*O*-d-glucoside (−37%), kaempferol-3-*O*-feruloyl-sophoroside-7-*O*-d-glucoside (−17%), and isorhamnetin-3-*O*-feruloyl-sophoroside-7-*O*-glucoside (−7%). In contrast, cooking resulted in the reduction of only 11% of flavonoid glycosides and hydroxycinnamic acid derivatives. A structure-specific degradation could be found as well. Complex flavonoid glycosides (kaempferol tri- and tetraglycosides) were degraded, whereas less complex ones (kaempferol diglycosides) such as kaempferol-3-*O*-caffeoyl-sophoroside (only in cooked samples), kaempferol-3-*O*-sinapoyl-sophoroside (+672 %), and kaempferol-3-*O*-feruloyl-sophoroside (+391 %) increased in their concentration. De-glycosylation of complex flavonoid glycosides seems to be the underlying mechanism during cooking of *B. carinata*. Caffeoylquinic acid, 3-p-coumarolyquinic acid as well as 5-p-coumaroylquinic acid were less affected by cooking.

With regard to carotenoids and chlorophylls, lutein, zeaxanthin, and β-carotene were present in the ethanolic extract as well as chlorophyll *a* and *b* (Table S3). Highest concentrations of chlorophylls, zeaxanthin, and β-carotene were found in the raw samples and lower concentrations in the cooked or fermented samples. Upon fermentation and cooking, the total carotenoid concentration declined by 75% and 40%, respectively. The largest losses were detected for lutein with 98% after fermentation and 42% after cooking. It should be noted that the zeaxanthin concentration increased during fermentation. While 23% of the initial chlorophyll was preserved by cooking, a complete degradation occurred during fermentation.

## Discussion

ALVs are increasingly recognized as potentially valuable for consumers’ health and are as such subject of global initiatives and scientific research, which aim to promote their beneficial effects [3]. Chronic exposure to aflatoxins and the associated disease burden is a persistent problem around the world. As developing countries are most severely afflicted [13,25], practical and economic approaches are essential to cope with this evident threat to human health.

In the present study, a protective potential against AFB1 could be demonstrated by ethanolic extracts from raw *B. carinata* plant material. This significantly blocked AFB1-mediated DNA strand breaks and demonstrated anti-oxidant as well as cytostatic potential in a human-derived liver cell model. It is well acknowledged that the *Brassicaceae* family possesses a variety of bioactive secondary plant metabolites, which are hypothesized to be responsible for some cancer-preventive observations [26,27]. It has been shown previously that ethanolic preparations of cabbage (*Brassica oleracea*) seeds protect against aflatoxicosis induced by aflatoxins in rats [28]. Furthermore, cabbage can significantly inhibit the binding of AFB1 to hepatic DNA and induced the activities of liver microsomal and cytosolic enzymes in rats [29]. Chinese cabbage (*Brassica chinensis*) also reduced the formation of AFB1-DNA adducts in liver cells and AFB1-induced liver tumors in mice [30]. Our results add to this picture by providing data on low-dose exposure in a human context and suggest that *B. carinata* is a promising candidate for dietary cancer chemopreventive measures.

However, ALVs are most commonly not eaten raw but processed for consumption. In Kenya this includes cooking [31,32] and fermentation [33]. In Western Kenya, traditional cooking of leafy vegetables sometimes is done for several hours, which is assumed to reduce the micronutrient content of the vegetables and affect bioactivity [31]. We demonstrated here, that processing of *B. carinata* leaves influences the content and composition of glucosinolates and their breakdown products, phenolic compounds, carotenoids, and chlorophylls. Interestingly, short cooking or fermentation did not abolish anti-genotoxicity or the anti-oxidant potential of the plant extract. Glucosinolates and isothiocyanates are well-acknowledged agents in cancer chemoprevention and thus have been hypothesized to play an important role in our study as well. Indeed, the glucosinolate sinigrin was detected in high amounts in extracts from raw, but less in extracts from cooked plant material and was not present in extracts from fermented plant tissue. Its bioactive form, allyl isothiocyanate, was only detected in the ethanolic extracts in very low concentrations. Hence, we have to conclude that the presence or absence of these compounds might not be of major relevance for the observed preventive effects.

The total phenolic content was clearly higher in the extracts from raw material of location A as compared to B and was subject to intensive decomposition during processing. The small hydroxycinnamic acid derivatives (caffeoylquinic acid, 3-p-coumarolyquinic acid, 5-p-coumaroylquinic acid) were the only ones found in approximately equal amounts in extracts from both locations. This concurs with a similar bioactivity, as observed in terms of anti-genotoxicity and anti-oxidant potency. Also, in contrast to the total phenolic content, these three compounds were not that much affected by processing, which again mirrors our findings in the bioassays. Various biological activities, including anti-oxidative and anti-carcinogenic activities, have been reported, for example, for caffeoylquinic acid before. It was found to exert radical scavenging and liver protective potential in human HepG2 cells [34], to inhibit methylazoxymethanol acetate-induced carcinogenesis in livers of hamsters [35] and 12-O-tetradecanoylphorbol-13-acetate-mediated tumor promotion in mice [36].

The significance of correlating analytical results with observed bioactivity remains debatable, because possible synergistic effects of the diverse bioactive compounds as well as activity of de-glycosylated compounds and decomposition products (e.g. complex flavonoid glycosides), need to be considered as well. For example, in kale the radical scavenging activity of polyphenols was reported to be stable although the original compound mixture was decomposed [37,38]. Synergy between compounds was also proposed in another study as only up to 24% of the observed anti-oxidant activity could be explained by the calculated sum of the individual phenolic compounds from red wine [39]. It also remains to be investigated how the single carotenoids and chlorophylls contribute to the observed effects.

## Conclusion

Similar to other *Brassica* plants, *B. carinata* is a rich source of potential cancer preventive compounds and ethanolic extracts of *B. carinata* leaves showed protective activity *in vitro*. Indeed, post-harvest processing in the form of fermentation or short boiling of the leaves of *B. carinata* has a significant impact on the content of secondary plant metabolites. However, the bioactivity of the leaves was not dramatically abrogated by the applied processing methodologies, but rather the protective effect was enhanced for some of the endpoints under study.

Based on the present *in vitro* findings, short time boiling could be considered an acceptable alternative to eating the raw leaves, as this does not appear to negatively impact the plant’s preventive potential. Furthermore, fermentation by *Lactobacillus plantarum* and *Lactobacillus fermentum* should also be considered, since this prevents spoilage and growth of pathogenic bacteria on the one hand [40,41], but also does not diminish bioactivity on the other. Therefore, consumption of *B. carinata* should be encouraged as part of dietary chemopreventive measures to combat prevalence of aflatoxin-induced diseases in general.

## Supplementary Material

Supplementary Data
