# Supplementary material for: The role of plant processing for the cancer preventive potential of Ethiopian kale (Brassica carinata)
Source: Food Nutr Res. 2017 Jan 31;61(1):1271527. doi: 10.1080/16546628.2017.1271527 (PMC5328379; doi:10.1080/16546628.2017.1271527)
Supplement: Supplementary Data [file zfnr_a_1271527_sm1720.docx]

**Supplementary material**

**Table S1.** **Glucosinolate and isothiocyanate content and composition of ethanolic *B. carinata* extracts.** Data are given in µg/mL (*n. d.* = not detectable).

|  | ***location A*** | | ***location B*** | |
| --- | --- | --- | --- | --- |
|  | ***raw*** | ***fermented*** | ***raw*** | ***cooked*** |
| ***glucosinolates (GLS)*** |  |  |  |  |
| 2-propenyl GLS / sinigrin | 1441.0 | *n. d*. | 1310.7 | 911.3 |
| 4-hydroxy-3-indolylmethyl GLS / 4-hydroxyglucobrassicin | 13.5 | *n. d* | 10.0 | 5.1 |
| 3-indolylmethyl GLS / glucobrassicin | 5.7 | *n. d* | 22.1 | 9.4 |
| 4-methoxy-3-indolylmethyl GLS / 4-methoxyglucobrassicin | 12.2 | *n. d* | 13.4 | 7.8 |
| 1-methoxy-3-indolylmethyl GLS / neoglucobrassicin | 1.3 | *n. d* | 5.5 | 4.1 |
| *total GLS* | 1473.7 | *n. d* | 1361.7 | 937.8 |
| ***isothiocyanates (ITC)*** |  |  |  |  |
| allyl ITC | 1.0 | 3.3 | 0.22 | *n. d.* |
| *total ITC* |  |  |  |  |

**Table S2.** **Phenolic compounds of ethanolic *B. carinata* extracts.** Data are given in µg/mL (*n. d.* = not detectable).

|  | ***location A*** | | ***location B*** | |
| --- | --- | --- | --- | --- |
|  | ***raw*** | ***fermented*** | ***raw*** | ***cooked*** |
| ***phenolic compounds*** |  |  |  |  |
| caffeoylquinic acid | 55.3 | 49.9 | 38.3 | 32.4 |
| caffeoyl-glycoside | 41.2 | 0.0 | 56.2 | 33.7 |
| 3-p-coumarolyquinic acid | 39.3 | 65.7 | 32.6 | 32.9 |
| 5-p-coumaroylquinic acid | 55.7 | 51.0 | 69.3 | 41.1 |
| quercetin-3-*O*-sophoroside-7-*O*-D-glucoside | 28.8 | 18.3 | 16.9 | *n. d.* |
| p-coumaroyl-glycoside | 88.9 | 50.3 | 39.4 | *n. d.* |
| kaempferol-3-*O*-sophoroside-7-*O*-D-glucoside | 367.0 | 127.3 | 107.8 | 26.1 |
| kaempferol-3-*O*-sophoroside-7-*O*-diglucoside | 49.7 | 10.7 | *n. d.* | *n. d.* |
| isorhamnetin-3-*O*-D-glucoside-7-*O*-D-glucoside | 133.6 | 22.3 | 40.9 | 60.9 |
| kaempferol-3-*O*-hydroxyferuoyl-sophoroside-7-*O*-D-glucoside | 1472.4 | 537.0 | 83.0 | 12.9 |
| kaempferol-3-*O*-caffeoyl-sophoroside-7-*O*-D-glucoside | 57.0 | 7.8 | 96.8 | 5.7 |
| isorhamnetin-3-*O*-hydroxyferuloyl-sophoroside-7-*O*-glucoside | 53.3 | 30.6 | 13.3 | 15.0 |
| isorhamnetin-3-*O*-caffeoyl-sophoroside-7-*O*-diglucoside | 27.2 | 13.3 | *n. d.* | 14.9 |
| isorhamnetin-3-*O*-hydroxyferuloyl-sophoroside-7-*O*-diglucoside | 122.7 | 25.6 | 21.9 | 10.7 |
| kaempferol-3-*O*-D-glucoside-7-*O*-D-glucoside | 181.4 | 34.8 | 28.1 | 8.4 |
| kaempferol-3-*O*-sinapoyl-sophoroside-7-*O*-diglucoside | 1195.5 | 212.3 | 215.0 | 20.5 |
| kaempferol-3-O-feruloyl-sophoroside-7-*O*-diglucoside | 94.8 | *n. d.* | 35.8 | *n. d.* |
| isorhamnetin-3-*O*-caffeoyl-sophoroside-7-*O*-D-glucoside | 1969.5 | 480.8 | 330.3 | 26.7 |
| kaempferol-3-*O*-sinapoyl-sophoroside-7-*O*-D-glucoside | 452.0 | 86.8 | 107.5 | 5.7 |
| kaempferol-3-*O*-feruloyl-sophoroside-7-*O*-D-glucoside | 49.4 | 40.8 | 8.9 | *n. d.* |
| kaempferol-3-*O*-coumaroyl-sophoroside-7-*O*-D-glucoside | 85.3 | 17.8 | 14.2 | 6.2 |
| isorhamnetin-3-*O*-sinapoyl-sophoroside-7-*O*-glucoside | 34.6 | 16.5 | 14.8 | 0.0 |
| isorhamnetin-3-*O*-feruloyl-sophoroside-7-*O*-glucoside | 19.8 | 18.4 | *n. d.* | *n. d.* |
| isorhamnetin-3-*O*-coumaroyl-sophoroside-7-*O*-glucoside | 37.2 | 15.5 | 14.4 | *n. d.* |
| isorhamnetin-3-*O*-gentiobioside | 25.0 | 0.0 | 42.9 | *n. d.* |
| kaempferol-3-*O*-hydroxyferuloyl-sophoroside | 753.3 | 15.9 | 85.8 | *n. d.* |
| isorhamnetin-3-*O*-sophoroside | 38.6 | *n. d.* | 33.0 | 27.3 |
| kaempferol-3-*O*-caffeoyl-sophoroside | 121.1 | 12.4 | *n. d.* | 194.0 |
| kaempferol-3-*O*-sophoroside7-sinapoyl-glucoside | 242.1 | 35.6 | *n. d.* | 114.9 |
| kaempferol-3-*O*-sinapoyl-sophoroside | 583.9 | 27.2 | 80.5 | 621.9 |
| kaempferol-3-*O*-feruloyl-sophoroside | 411.6 | 18.6 | 59.9 | 294.7 |
| quercetin-3-*O*-disinapoyl-triglucoside-7-*O*-D-glucoside | 76.8 | 38.1 | *n. d.* | *n. d.* |
| kaempferol-3-*O*-disinapoyl-triglucoside-7-*O*-D-glucoside | 1063.0 | 285.8 | 164.2 | 13.1 |
| disinapoyl-gentiobioside | 300.9 | 150.1 | 50.4 | 33.7 |
| sinapoyl-feruloyl-gentiobiose | 70.1 | 44.8 | 41.8 | 39.3 |
| trisinapoyl-gentiobioside | 301.9 | 114.5 | 62.8 | 89.4 |
| disinapoyl-feruloyl-gentiobiose | 105.8 | 55.4 | 46.1 | 44.5 |
| *total phenolic compounds* | 10805 | 2732 | 2053 | 1827 |

**Table S3.** **Carotenoid and chlorophyll content and composition of ethanolic *B. carinata* extracts.** Data are given in µg/mL (*n. d.* = not detectable).

|  | ***location A*** | | ***location B*** | |
| --- | --- | --- | --- | --- |
|  | ***raw*** | ***fermented*** | ***raw*** | ***cooked*** |
| ***carotenoids*** |  |  |  |  |
| lutein | 32.1 | 0.8 | 41.6 | 24.0 |
| zeaxanthin | 1.1 | 7.6 | 5.4 | 4.3 |
| β-carotene | 0.2 | 0.1 | 0.1 | 0.1 |
| *total carotenoids* | 34.2 | 8.4 | 47.1 | 28.3 |
| ***chlorophylls*** |  |  |  |  |
| chlorophyll a | 133.5 | *n. d.* | 272.63 | 46.9 |
| chlorophyll b | 71.3 | *n. d.* | 102.5 | 40.1 |
| *total chlorophylls* | 204.8 | *n. d.* | 375.1 | 87.0 |
